# Cognitive control and its impact on recovery from aphasic stroke

**DOI:** 10.1093/brain/awt289

**Published:** 2013-10-24

**Authors:** Sonia L.E. Brownsett, Jane E. Warren, Fatemeh Geranmayeh, Zoe Woodhead, Robert Leech, Richard J. S. Wise

**Affiliations:** 1 Cognitive, Clinical and Computational Neuroimaging Group, Imperial College, Hammersmith Hospital, London, W12 0NN, UK; 2 Department of Cognitive, Perceptual and Brain Sciences, Division of Psychology and Language Sciences. University College London, UK; 3 Wellcome Trust Centre for Neuroimaging, University College London, UK

**Keywords:** aphasia, salience, cingulate, executive, functional MRI

## Abstract

Aphasic deficits are usually only interpreted in terms of domain-specific language processes. However, effective human communication and tests that probe this complex cognitive skill are also dependent on domain-general processes. In the clinical context, it is a pragmatic observation that impaired attention and executive functions interfere with the rehabilitation of aphasia. One system that is important in cognitive control is the salience network, which includes dorsal anterior cingulate cortex and adjacent cortex in the superior frontal gyrus (midline frontal cortex). This functional imaging study assessed domain-general activity in the midline frontal cortex, which was remote from the infarct, in relation to performance on a standard test of spoken language in 16 chronic aphasic patients both before and after a rehabilitation programme. During scanning, participants heard simple sentences, with each listening trial followed immediately by a trial in which they repeated back the previous sentence. Listening to sentences in the context of a listen–repeat task was expected to activate regions involved in both language-specific processes (speech perception and comprehension, verbal working memory and pre-articulatory rehearsal) and a number of task-specific processes (including attention to utterances and attempts to overcome pre-response conflict and decision uncertainty during impaired speech perception). To visualize the same system in healthy participants, sentences were presented to them as three-channel noise-vocoded speech, thereby impairing speech perception and assessing whether this evokes domain general cognitive systems. As expected, contrasting the more difficult task of perceiving and preparing to repeat noise-vocoded speech with the same task on clear speech demonstrated increased activity in the midline frontal cortex in the healthy participants. The same region was activated in the aphasic patients as they listened to standard (undistorted) sentences. Using a region of interest defined from the data on the healthy participants, data from the midline frontal cortex was obtained from the patients. Across the group and across different scanning sessions, activity correlated significantly with the patients’ communicative abilities. This correlation was not influenced by the sizes of the lesion or the patients’ chronological ages. This is the first study that has directly correlated activity in a domain general system, specifically the salience network, with residual language performance in post-stroke aphasia. It provides direct evidence in support of the clinical intuition that domain-general cognitive control is an essential factor contributing to the potential for recovery from aphasic stroke.

## Introduction

Recovery from aphasic stroke can be both variable and unpredictable. The size of the lesion and the age of the patient only account for ∼40% of this variance (Lazar *et al.*, 2008). Irrespective of lesion volume, further factors influencing the capacity for recovery may include the exact location of the lesion (Kertesz *et al.*, 1979; Heiss *et al.*, 1999; Plowman *et al.*, 2012; Schofield *et al.*, 2012) or the initial type or severity of the aphasia (Kertesz and McCabe, 1977; Pedersen *et al.*, 2004; Bakheit *et al.*, 2007). Given the limited knowledge about the systems neuroscience of recovery and rehabilitation after focal brain injuries, it has been one of the goals of functional neuroimaging research to afford insight into the brain networks supporting recovery from aphasia (Musso *et al.*, 1999; Leff *et al.*, 2002; Abo *et al.*, 2004; Fernandez *et al.*, 2004; Naeser *et al.*, 2004; Price and Crinion, 2005; Saur *et al.*, 2006, 2010; Warren *et al.*, 2009; see also a review by Meinzer *et al.*, 2011).

However, consensus has been limited (Hamilton *et al.*, 2011). Some authors have argued that successful recovery depends on the function of intact perilesional tissue (Heiss *et al.*, 1999; Warburton *et al.*, 1999; Rosen *et al.*, 2000). Others have proposed that a ‘laterality shift’ of language functions from the left to the right hemisphere may occur, either immediately after the ictus with a subsequent shift back to the left hemisphere (Saur *et al.*, 2006) or as a permanent reorganization (Weiller *et al.*, 1995; Musso *et al.*, 1999; Thompson *et al.*, 2000; Leff *et al.*, 2002; Raboyeau *et al.*, 2008). Yet others have concluded that the contribution of ‘homologous’ language regions in the right hemisphere is unrelated to, or may even inhibit, recovery in the left hemisphere (Rosen *et al.*, 2000; Thiel *et al.*, 2001; Blank *et al.*, 2003; Naeser *et al.*, 2004, 2005; Winhuisen *et al.*, 2007) or only contribute to recovery in the chronic stage (Mimura *et al.*, 1998; Richter *et al.*, 2008).

A common, but not universal, assumption is that the behavioural tasks are activating domain-specific language processes, and if in patients they are located in regions not observed in healthy participants performing the same task on the same stimuli, then language processes have become reorganized to atypical sites. However, many language tasks given to participants in functional imaging environments are rarely encountered in everyday life. As we have argued previously (Wise, 2003), at least some of the activity observed in patients may relate to greater engagement of normal and intact domain-general executive and attentional networks as the patients struggle with the task, rather than to language processing *per se*. Price and Friston (1999) addressed this issue almost 15 years ago, arguing that patients should be given ‘tasks they can perform’. In practice this is often difficult to achieve, as patients rarely make a complete recovery, and even if the patients achieve a performance that matches the healthy participants, it may be at the expense of greater ‘cognitive effort’.

The alternative is to make things more difficult for the healthy participants. The present study investigated activity in a group of 16 chronic patients with post-stroke aphasia with a task that healthy participants can perform with ease, namely listen to a short sentence of clear speech and then repeat it back after a few seconds delay. Task difficulty was increased for the healthy participants by presenting them with trials in which they were required to listen to similar sentences but the speech presented had much of the acoustic information removed (3-channel noise-vocoded speech) (Shannon *et al.*, 1995). Analysis and interpretation of the data was made in the light of new knowledge about the balance between activity within the default mode network, typically active during ‘rest or passive’ states, and activity in the salience (cingulo-opercular) and central executive (fronto-parietal) networks, active during attention to external stimuli and task-related performance on these stimuli. There is now abundant evidence that the activity over time in the default mode network and the salience/central executive networks are anticorrelated (Raichle *et al.*, 2001; Greicius *et al.*, 2003; Greicius and Menon, 2004) and further, that pathological states may interfere with the balance between the interoceptive (default mode) and exteroceptive (salience/central executive) networks (Anticevic *et al.*, 2012; Bonnelle *et al.*, 2012). Of particular note is that the opercular component of the salience network is located in the anterior insular and frontal opercular cortices, bilaterally (Menon and Uddin*,* 2010).

In a typical language study, these regions may be only too readily labelled as Broca’s area and its homologue. This carries the implicit assumption that activity observed in the frontal opercula is related to language-specific processing, when it is as feasible that it is associated with domain-general, task-dependent processes. Some studies have implicated this region in cognitive control of language rather than language *per se* in both imaging studies of healthy volunteers (Snyder *et al.*, 2007; see also Novick *et al.*, 2010) and behavioural studies of patients with inferior frontal gyrus lesions (Tseng *et al.*, 1993; Novick *et al.*, 2009). Fridriksson and Morrow (2005) suggested that their observed correlation between increases in activity in inferior frontal gyrus and task performance may reflect domain–general processes, such as increased task difficulty because of greater working memory load. Indeed some authors have suggested that language is not lost in aphasia but impaired linguistically specialized attentional system that is vulnerable to competition instead induces language deficits observed (Hula *et al.*, 2008). The issue about whether activity in ‘classic’ Broca’s area may be language-specific or related to domain-general cognitive control has been addressed further in a study by Fedorenko *et al.* (2012). In this functional MRI study on normal participants, closely adjacent voxels within both left Brodmann areas (BA) 44 (pars opercularis) and 45 (pars triangularis) responded to a language task or to multiple tasks, verbal and non-verbal. The authors’ conclusion was that Broca’s area contains domain-specific language subregions intermingled with others that respond to a broad range of tasks and domains.

The first hypothesis under test in this study was that the pattern of activity during the listen-and-prepare-to-repeat trials (ListTrials) in the patients listening to clear speech would be equivalent to that in the healthy participants during ListTrials for 3-channel vocoded speech (ListVoc), both in terms of activation of the salience/central executive networks and deactivation of the default mode network. In contrast, ListTrials for clear speech in the healthy participants were expected to result in less activation of the salience/central executive networks and a corresponding reduced deactivation of the default mode network. Once this analysis confirmed the increased ‘cognitive effort’ that the patients needed to apply to clear speech was equivalent to that observed in healthy participants confronted with 3-channel noise vocoded speech, the second aim was to investigate whether domain-general activity would be variable across patients, and whether this variability would correlate with performance on a test that assesses language use, namely picture description. The dorsal anterior cingulate cortex and adjacent part of the midline superior frontal gyrus (dACC/SFG) was chosen as the target domain-general region as it is the one component of the salience and central executive networks that lies within anterior cerebral artery territory, whereas aphasic strokes are usually the consequence of infarction within middle cerebral artery territory. Additionally, functional imaging studies on healthy participants have shown that internally generated speech activates the dACC/SFG (Braun *et al.*, 1997; Blank *et al.*, 2002; Geranmayeh *et al.*, 2012).

A further area of investigation was the influence of behavioural training on auditory discrimination of normal speech in the patients. In parallel, the healthy participants received training on discriminating noise-vocoded speech. Although subtle behavioural changes were observed (in press), these changes were not mirrored by any apparent change on the functional imaging data acquired before and after training in either the patients or the healthy participants. For the sake of brevity, the analyses that resulted in this null result are not included here.

The strategy, therefore, was to elicit activity in the dACC/SFG by a task that can be readily implemented in an functional MRI study, and then relate it to a task outside the scanner that more transparently reflects everyday use of language, namely descriptive speech, but which is difficult to implement in a scanning environment in patients with residual aphasia.

## Materials and methods

### Participants

**Figure 1:**
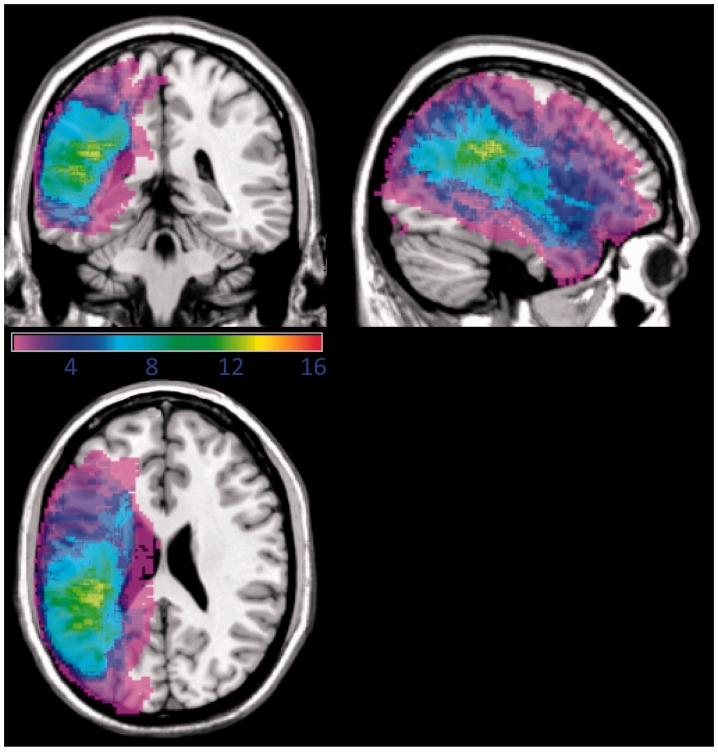
Overlay of the lesion distribution in the 16 patients with post-stroke aphasia. Projections are rendered onto a single-subject brain template. The colour code represents the absolute number of participants with a lesion in a given voxel (range: 1 shown in purple to 16 shown in red).

Eighty-eight right-handed patients with persistent post-stroke aphasia were screened. For a variety of reasons (Supplementary material), only 16 patients (five females, mean age 60 years; range 37–84 years) completed the study. The mean duration of formal education was 15 years (range 10–18 years). All patients were at least 6 months post-stroke (mean 4 years, range 6 months to 11 years), at a time when further spontaneous recovery is likely to be negligible (Lendrem *et al.*, 1985). All patients had a lesion involving the left temporal and parietal lobes, and four patients had a lesion extending into the frontal lobe but not involving anterior cerebral artery territory (Fig. 1). All patients presented with an auditory comprehension and repetition deficit. The patients’ comprehension was sufficient for them to give informed consent and to understand what was required of them. Most patient’s production skills were sufficient to allow them to attempt to repeat short sentences, although in two patients only single words were produced when attempting to repeat the sentences. No patients presented with a pure apraxia of speech. Other inclusion criteria were no history of other neurological illness, no sinistrality and at the time of participation they were not receiving speech and language therapy.

Healthy participants (control subjects) had no history of neurological illness, no sinistrality, no history of developmental language impairment, no contraindications to MRI and English as the first language. Seventeen participants completed the study (11 females; mean age 60 years; range, 25–82 years) with a mean duration of formal education of 15 years (range 10–20 years).

Ethical approval for the study was granted by Hammersmith, Queen Charlotte’s and Chelsea Research Ethics Committee, London, UK.

### Functional magnetic resonance imaging

Patients participated in three scanning sessions and healthy participants in two sessions. The healthy participants received behavioural training on discriminating phonetic contrasts within noise-vocoded speech for the 2 weeks between their two scans. Patients participated in the therapy programme for 4 weeks between their second and third scans. The scanning protocol was identical for each session but used a different set of stimuli.

MRI data were obtained on a Philips Intera 3.0 T scanner using dual gradients, a phased array head coil, and sensitivity encoding with an undersampling factor of two. Functional magnetic resonance images were obtained using a T_2_*-weighted, gradient-echo, echoplanar imaging (EPI) sequence with whole-brain coverage (repetition time, 8.0 s; acquisition time, 2.0 s; echo time, 30 ms; flip angle, 90°). Quadratic shim gradients were used to correct for magnetic field inhomogeneities within the anatomy of interest. Speech output was recorded using a magnetic resonance-compatible microphone attached to ear-defending headphones to assess task performance. Padding around the headphones was also used to minimize head movement. Participants were able to hear their own speech, although as the earphones were noise reducing, the balance between air conduction and bone conduction was altered. At the first scanning session, a high-resolution T_1_-weighted structural scan was obtained in both healthy participants and patients between two separate functional MRI runs.

**Figure 2:**
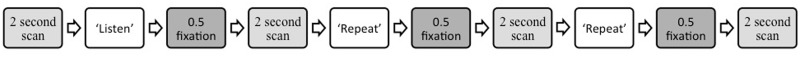
Scanning paradigm in functional MRI for healthy volunteers.

**Figure 3:**
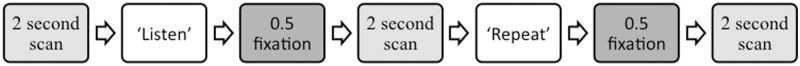
Scanning paradigm in functional MRI for subjects with aphasia.

A ‘sparse’ functional MRI design was used to minimize movement- and respiratory-related artefacts associated with speech studies. Assuming that some patients would also have impaired auditory stream segregation, the use of ‘sparse’ sampling also removed the distracting effect of background scanner noise during the ListNorm trials. Tasks were performed over 5.5 s while a visual task prompt was displayed. The disappearance of that prompt and the appearance of a fixation crosshair signalled to the subject to cease the task. Two seconds of data acquisition commenced 0.5 s later, during which the fixation crosshair remained present. This cycle was repeated for the duration of each run (Figs 2 and 3).

### Stimuli

In an attempt to simulate the difficulties in comprehension seen in the patient group, and as used in a previous study on post-stroke aphasia (Sharp *et al.*, 2004), the healthy participants had trials on 3-channel noise-vocoded speech functional MRI (Shannon *et al.*, 1995). Comprehension of noise-vocoded speech depends largely on the number of frequency channels, and preliminary behavioural testing indicated that three frequency channels would produce a level of repetition impairment equivalent to that encountered in the patient group. Bamford-Kowal-Bench sentences (Bench *et al.*, 1979) were used as these have a low sentence-end predictability, which would limit the amount of top-down semantic processing being used to discriminate and understand the sentences (e.g. ‘He is buying some bread’, where the final word could be any item that may be purchased). They are also sentences without complex syntax.

### Scanning paradigms

The scanning paradigm for the healthy participants involved a ‘listen-repeat-repeat’ design across two runs. There were 140 trials in each run (Fig. 2). Participants were presented with Bamford-Kowal-Bench sentences, to which they were required to listen and then repeat in two subsequent trials. Each run consisted of 20 sentences presented using normal clear speech stimuli, and 20 using normal stimuli that had been noise-vocoded. The purpose of the two repeat trials was to observe the effects of masking auditory feedback with white noise on one of the two repetition trials. As will become apparent later, all the signal analysed for this study was in the listening trials, and so the effect of auditory masking during repetition is not discussed further here. A low-level auditory baseline (20 trials spaced irregularly between ‘listen-repeat-repeat’ patterns) of listening to segmented broadband noise bursts (white noise) matched in duration to sentence stimuli was also used. White noise is a complex sound but contains no spectrotemporal structure.

A similar, yet simpler and shorter, paradigm was given to patients with aphasia. Shortening the duration of scanning made the procedure more acceptable to disabled patients. They had 84 trials in each run, where they were presented with a Bamford-Kowal-Bench sentence presented as normal speech, they were required to listen to each sentence and then repeat it in the subsequent trial (Fig. 3). The same low-level auditory baseline of listening to segmented broadband noise bursts was included.

### Univariate whole-brain analyses

Univariate analyses were carried out within the framework of the general linear model using FEAT (FMRI Expert Analysis Tool) Version 5.98, part of FSL (FMRIB's Software Library, www.fmrib.ox.ac.uk/fsl). The following image preprocessing steps were applied: realignment of EPI images for motion correction using MCFLIRT; non-brain removal using BET (Brain Extraction Tool); spatial smoothing using a 6 mm full-width half-maximum Gaussian kernel; grand-mean intensity normalization of the entire 4D data set by a single multiplicative factor; and high pass temporal filtering (Gaussian-weighted least-squares straight line fitting, with sigma = 50 s) to correct for baseline drifts in the signal. Time-series statistical analysis was carried out using FILM (FMRIB’s Improved Linear Modelling) with local autocorrelation correction. Registration to high resolution structural and Montreal Neurological Institute (MNI) standard space images (MNI 152) were carried out using FMRIB's Linear Image Registration Tool (FLIRT) and cost-function masking (described below) was applied to patients’ structural scans in order to avoid the known problem of stretching normal tissue to fill the infarct during standard registration (Brett *et al.*, 2001).

The combination of the different runs at the individual subject level was analysed using a fixed-effects model. Individual first-level design matrices were created, modelling the different behavioural conditions. Contrast images of interest were produced from these individual analyses and used in the second-level higher analysis. Higher-level between-subject analysis was carried out using a mixed-effects analysis with the FLAME (FMRIB's Local Analysis of Mixed Effects) tool, part of FSL. Final statistical images were corrected for multiple comparisons using Gaussian Random Field-based cluster inference with a height threshold of *Z* > 2.3 and a corrected significance threshold of *P* < 0.05.

### Lesion masking

Individual 3D were hand drawn on T_1_-weighted templates for each slice using FMRIB Software Library image viewer (FSLView). A lesion mask was then created by binarizing the image and then inverting it. The patients’ functional MRI scans were registered to their structural T_1_ using FLIRT with 6 degrees of freedom. Next, the patient’s structural image was registered to the standard MNI anatomical template using FLIRT with 12 degrees of freedom, with the binary inverted lesion image as an input-weighting mask to down-weight the influence of the damaged area on the registration solution and so avoid the distortion associated with normalization of brains with sizeable infarcts. The two resulting transformation matrices (functional to structural and structural to standard) were then concatenated and applied to the functional data to achieve functional to standard registration.

### Comparison between groups

The individual contrasts versus baseline were carried out at the first level for both patients and healthy participants. The results were passed on to the second level, which used a fixed effects model to combine the two runs for each scanning session. At the third level a group mixed-effects analysis modelled an independent samples *t*-test comparing patient and control groups.

### Region of interest analysis

A region of interest analysis was carried out to relate activity in the medial part of the salience (cingulo-opercular) network (dACC/SFG) with the aphasic patient’s performance on an off-line test of speech production, namely picture description. To provide an unbiased way of extracting data, the theoretically motivated region of interest was defined by multiplying probabilistic anatomical masks from the FSL Harvard-Oxford Cortical Structural Atlas with functional activity observed in healthy participants. The region of interest mask was re-registered to the same space as individual preprocessed functional data from the univariate analysis. Using FSL FEATQuery within the region of interest, effect sizes for the different conditions and different runs were calculated for each individual. The mean across the two runs was then calculated to provide a mean effect size for each session. Repeated measures analysis of variance (ANOVA), bivariate correlations and *t*-tests were used to analyse the region of interest data using SPSS (IBM Corp). Multiple regression analysis was used to regress out the effects of lesion volume and patient’s age from the correlation analysis, with the picture description score as the independent variable and dACC/SFG activation, lesion volume and age as the dependent variables.

### Scoring in-scanner responses

Three scores for each participant’s spoken responses during scanning were calculated: a semantic score; an articulation score and a combined semantic and articulation score. A semantic score of five points was awarded if the whole sentence was repeated correctly, four points if all the content words were produced but one or more function words were omitted, three if >50% of the content words were produced, two if <50% of the content words were produced, one if a single appropriate word was attempted and zero if there was no response or fillers only. The same scoring system was used for the articulation score: five points if the whole sentence was correctly articulated; four points if all the content words were correctly articulated but some function words or inflections were incorrect or omitted; three if >50% of the sentence was correctly articulated; two if <50% of the content words were produced, one if a single appropriate word was attempted; and zero if there was no response or fillers only. The mean of the semantic and articulation score was calculated to produce a combined score. The reason for scoring like this was important to account for any speech errors, both articulatory and phonological, produced by the patients while producing speech (e.g. one patient produced the sentence ‘The clown had a funny face’ as ‘the /tlown/ had a funny /sais/’. Clearly, all the keywords are produced, but not if accurate articulation was required to score the keyword. Likewise, scoring 100% for producing all the keywords would not capture the fact that the patient did not produce the sentence normally.

### Behavioural training

#### Patients

The patients with aphasia received behavioural therapy, delivered as a home-based computer program. The computer program consisted of five therapeutic auditory discrimination and repetition tasks. The study was designed to progress patients through higher levels of difficulty. This was delivered over 4 weeks, between the second and third scanning sessions. The primary aim of the rehabilitation programme was to investigate behavioural retraining of auditory (phonological) discrimination and these results (along with those for healthy participants) are being prepared for separate publication, but any changes in the functional MRI signal as the result of training were included in the analyses of the imaging data presented here. The stimuli and tasks used for the training programme for healthy participants were identical to that for the patients as discussed above, but all stimuli were 3-channel noise-vocoded. Healthy volunteers completed 2 weeks of training and patients 3 weeks of therapy.

### Picture description

Before each scanning session, participants with aphasia completed a picture description task describing a complex picture for 1 min. The picture was taken from the comprehensive aphasia test (Swinburn *et al.*, 2004). The picture descriptions were transcribed and scored according to criteria in the comprehensive aphasia test manual by two of the authors (S.L.E.B. and F.G.). This comprised scores of the sum of appropriate information-carrying words minus inappropriate information-carrying words, syntactic variety, grammatical well-formedness, and speed of speech production.

## Results

### Behavioural results

#### Patients

Despite wide interindividual variability, the patients’ performance on the repeating of normal speech trials (repeat normal spoken sentence stimuli) correlated significantly (using the combined score for articulation and semantics) between scanning sessions one and two (Pearson’s *r* = 0.88, *P* < 0.001); between sessions two and three (*r* = 0.84, *P* < 0.001); and between sessions one and three (*r* = 0.94, *P* < 0.001). Similarly, paired *t*-tests demonstrated no significant differences between any sessions using any of the three measures (*P* > 0.1).

#### Healthy participants

Predictably, the healthy participants were better at repeating after listening to normal speech trials (ListNorm) than listening to vocoded speech trials (ListVoc). Before training, using the combined semantic and articulation score as the measure: *t*(15) = 13; two-tailed; *P* < 0.001; 95% confidence interval (CI) = 42.6–59.4. After training: *t*(16) = 11.6, two-tailed; *P* < 0.001; 95% CI = 28.2–40.7. The training programme, aimed at improving auditory perception and lexical recognition of 3-channel noise-vocoded speech, demonstrated a significant difference between pre- and post-training repeat vocoded spoken sentence stimuli trials for all behavioural measures (articulation, semantic and the combined score). Thus, on the combined score, the mean percentage improvement on noise-vocoded stimuli was 15.5%, an improvement that was significant: *t*(15) = 6.44, *P* < 0.001, two-tailed; 95% CI = 10.4–20.6. Predictably, there was no difference on repeat normal spoken sentence stimuli trials (mean = 1.1%) as the result of training. Performance was at ceiling at both time-points: *t*(15) = 1.5, *P* > 0.1, two-tailed; 95% CI = −0.4–2.5.

**Figure 4:**
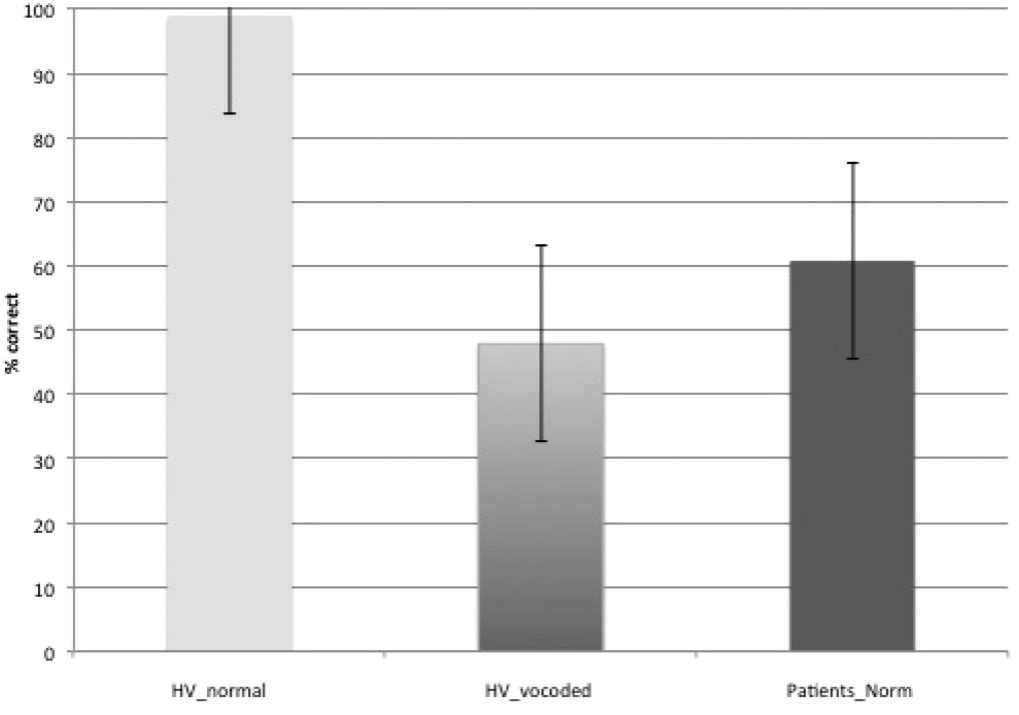
Bar chart showing the mean percentage accuracy (with standard error) on repetition accuracy during scanning across both intelligibility conditions in the healthy volunteer (HV) group and in the patient group on normal speech.

When comparing the combined scores (articulation and semantics) on repeat normal spoken sentence stimuli trials in the patients with the repeat vocoded spoken sentence stimuli in the healthy participants, an independent-samples *t*-test with equal variances not assumed, showed there was no difference between groups [*t*(22.7) = 1.7, *P* = 0.1]. Therefore, the aim of making the task of approximately comparable difficulty in patients and healthy participants was achieved (Fig. 4).

### Imaging results

#### Patients

The patients had three scans compared to the healthy participants’ two scans. This was to enable the patient population, who were expected to find the scanning experience more stressful than the normal population, to acclimatize to the experience before obtaining pre- and post-training scan data. A repeated measures analysis was carried out to investigate functional differences between scanning sessions, using contrasts of ListNorm with ListWhite for each session. Assuming that activity in response to ListWhite was stable across sessions, there was no greater activity in cortical or subcortical grey matter regions in response to ListNorm during session one relative to either two or three or in session two relative to three.

**Figure 5:**
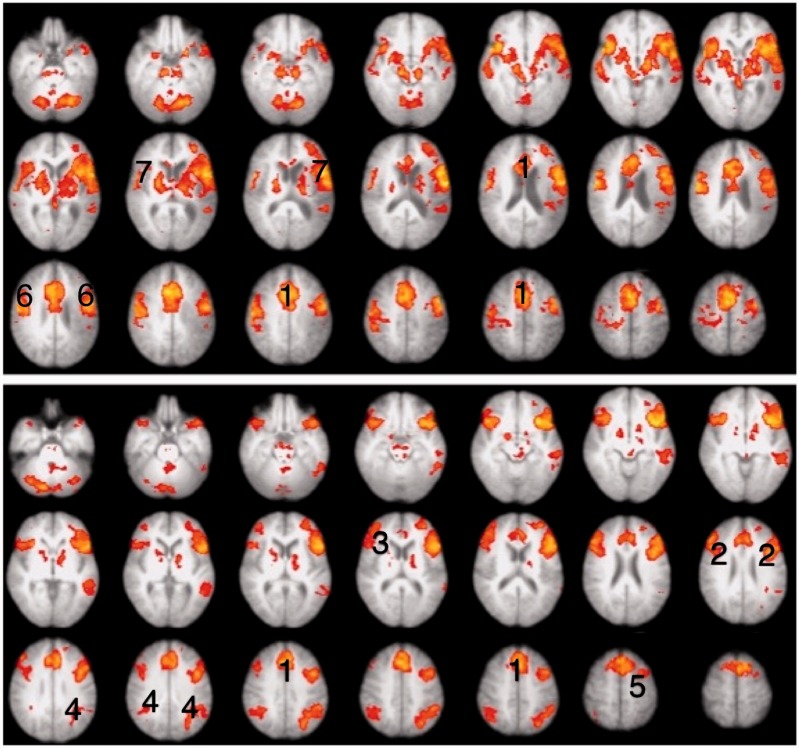
Thresholded *Z*-statistic images for the contrasts of the *top* panel: listening to normal stimuli versus repeating normal stimuli in participants with aphasia (mean of both scanning sessions). *Bottom* panel: listening to vocoded stimuli versus listening to normal stimuli in healthy volunteers (mean of both sessions). All images are thresholded using clusters determined by *Z* > 2.3 and a (corrected) cluster significance threshold of *P* = 0.05. Numbers identify activity within (1) the dACC/SFG, (2) inferior frontal gyrus and adjacent anterior insular cortex, (3) dorsolateral prefrontal cortex, (4) dorsal inferior parietal cortex and adjacent lateral intraparietal sulcus (dorsal inferior parietal cortex and adjacent lateral intraparietal sulcus) and (5) middle frontal gyrus.

Excluding session one, thereby making an equivalent comparison with the data on the healthy participants, a Task (listen and repeat) × Session (pre- and post-training) ANOVA was performed. There was no Task × Session interaction. The main effect of Task revealed extensive activation in bilateral premotor (lateral and medial) and primary somatosensory-motor cortex, the length of both superior temporal gyrus from the plana temporale to the temporal poles, dACC/SFG and bilateral inferior frontal gyrus and adjacent anterior insular cortex [the salience (cingulo-opercular) network], bilateral dorsolateral prefrontal cortex and right dorsal inferior parietal cortex and adjacent lateral intraparietal sulcus [the central executive (fronto-parietal) network], posterior midline cortex and posterior right inferior parietal cortex. Small areas of the left posterior middle temporal gyrus and left parietal operculum were also activated in those patients in whom those regions remained intact. Subcortical regions included bilateral basal ganglia (but not the anterior striatum), the thalami and bilateral paravermal cerebellum. *Post hoc* comparisons revealed that all regions were more active in the ListNorm relative to the repeat normal spoken sentence stimuli trials (Fig. 5), except the posterior midline cortex (posterior cingulate cortex and adjacent precuneus) and right inferior parietal cortex, components of the default mode network (minus the infarcted left inferior parietal cortex), were evident in the reverse contrast. Therefore, it appears that subvocal rehearsal in the ListNorm trials was present in addition to activation in high-order cognitive control networks, with less ‘cognitive effort’ being exerted when it came to repetition as there was less deactivation of the default mode network during these trials. The additional contrast of ListWhite versus ListNorm also revealed areas associated with the default mode network that overlapped with the regions evident in the contrast of repeat normal spoken sentence stimuli with ListNorm.

A main effect of session revealed a small area of activation in the precuneus. Dividing the two tasks, paired *post hoc t*-tests to investigate between session effects, relative to ListWhite, demonstrated no differences between the two sessions for either task.

#### Healthy participants

**Figure 6:**
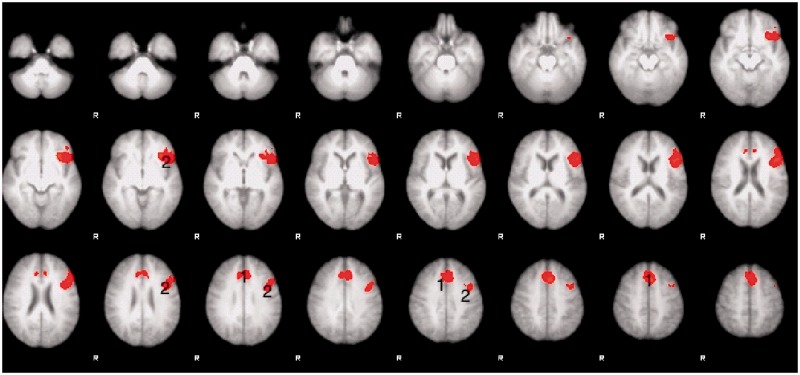
Thresholded *Z* statistic images for the Task × Intelligability interaction found in healthy volunteers. All images are thresholded using clusters determined by *Z* > 2.3 and a (corrected) cluster significance threshold of *P* = 0.05. Numbers identify activity within (1) the dACC/SFG and (2) inferior frontal gyrus and adjacent anterior insular cortex. R = right.

The initial analysis was a whole-brain Task (listening and repeating) × Intelligibility (clear and 3-channel noise-vocoded speech) × Session (before and after training) ANOVA. A Task × Intelligibility interaction was observed in the left inferior frontal gyrus (including pars opercularis and triangularis) and anterior insula (inferior frontal gyrus and adjacent anterior insular cortex), extending up into the middle frontal gyrus, and in the dACC/SFG (Fig. 6). *Post hoc* contrasts between the various conditions demonstrated that the signal in dACC/SFG was activated by the ListVoc trials relative to both ListNorm and repeat vocoded and normal spoken sentence stimuli. The contrast of ListVoc with List Norm clearly demonstrated activity throughout both the salience and central executive networks (Fig. 5). The reverse contrast, of the easier with the more difficult listening condition, demonstrated the default mode network. Therefore, listening to normal speech in the patients and listening to noise-vocoded speech in the healthy participants were equivalent in terms of deactivating the default mode network. There were no voxels that survived the statistical threshold for the Session × Task, Session × Intelligibility, and Session × Task × Intelligibility interactions. Therefore, and as prefaced in the ‘Introduction’ section, any effects of the training programme on brain activity were not apparent in this univariate ANOVA.

#### Between group comparison

A direct comparison between the patients and healthy participants was carried out to investigate both effects of a lesion on the ListNorm trials, and also similarities because of simulating the functional effects of the lesion by using noise-vocoded speech in the healthy participants.

To evaluate differences in processing clear speech between patient and normal groups, a mixed-effects, independent samples *t*-test (ListNorm contrasted with ListWhite for both patients and healthy participants) was carried out. The contrast of healthy participants > patients demonstrated greater activity within the default mode network, including the precuneus, posterior cingulate cortex, and medial prefrontal cortex. Additional greater activity was observed in left ventral prefrontal cortex and right medial planum temporale at the first (pre-training) session but not the second (post-training). The reverse contrast of patients > healthy participants demonstrated greater activity in the salience (cingulo-opercular) network for both sessions.

To evaluate differences in processing clear and vocoded speech in the patient and normal groups, respectively, an additional mixed-effects, independent samples *t*-test, (using ListNorm contrasted with ListWhite for patients and ListVoc contrasted with ListWhite for the healthy participants) was carried out. These comparisons revealed no differences in either the pre- or post-training sessions.

### Summary of whole-brain analyses

**Figure 7:**
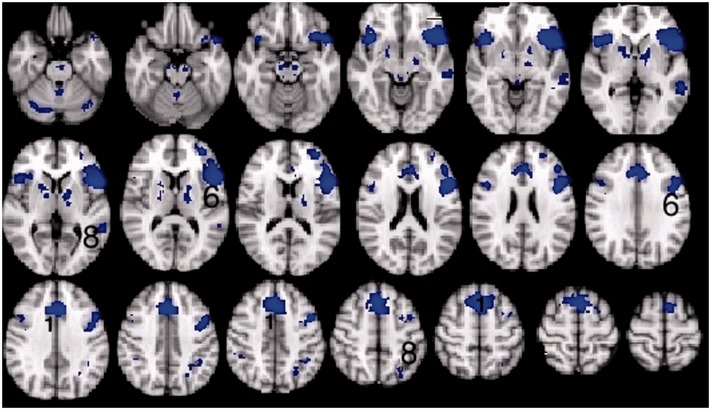
Thresholded *Z* statistic images for the contrasts of listening to vocoded stimuli versus listening to normal stimuli in healthy volunteers (mean of both sessions) multiplied by the contrast of listening to normal stimuli versus listening to white noise in patients (mean of sessions 2 and 3). All images are thresholded using clusters determined by Z > 2.3 and a (corrected) cluster significance threshold of *P* = 0.05. Numbers identify activity within (1) the dACC/SFG and (6) inferior frontal gyrus and adjacent anterior insular cortex, (8) dorsal inferior parietal cortex and adjacent lateral intraparietal sulcus.

The results demonstrated that increased signal was evident in the trials in which participants listened and prepared to repeat the Bamford-Kowal-Bench sentences, whereas the repeating trials conveyed no useful additional signal. When healthy participants listened to noise-vocoded speech, but not normal speech, and when patients listened to normal speech, there was deactivation of the default mode network, and activity in the salience/central executive networks was equivalent in the two groups (Fig. 7). There was no evidence of anatomical shifts of domain-specific language processing. In addition, the effects of training in both groups produced minor differences in activity within these networks.

### Region of interest analysis

**Figure 8:**
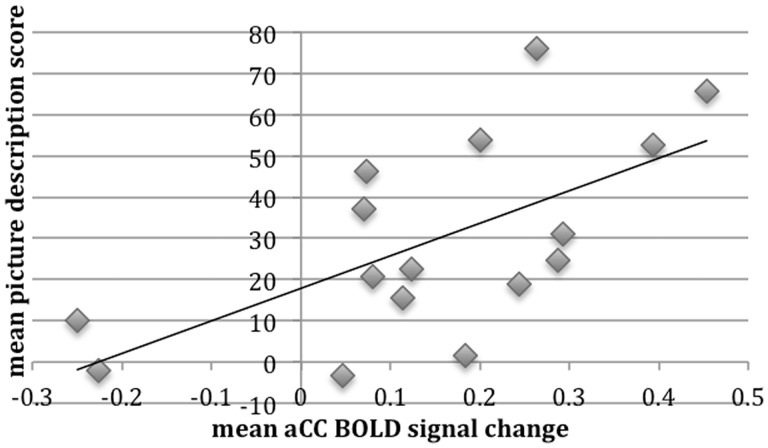
Correlation between patients’ mean picture description scores and mean dACC/SFG percent signal change across all three sessions. BOLD = blood oxygen level-dependent.

Based on the results from the whole-brain analyses, with activity in high-order cognitive cortices increasing with difficulty (as the result of stroke in the patients and manipulated perceptual difficulty in the healthy participants) a region of interest analysis was performed. The dACC/SFG region was chosen as it is located in anterior cerebral artery territory, and therefore outside the vascular territory of infarction in the patients. Activated voxels in this region from the contrast of ListVoc with ListNorm in the healthy participants was multiplied by a standard anatomical template for the cingulate cortex and adjacent SFG. Having defined this functional-anatomical region in the normal group, this region of interest was applied to the data from the patients. Activity in dACC/SFG in the patients was then regressed against their off-line performance on the picture description task. There is abundant evidence in the literature that the internal generation of narrative speech activates the dACC/SFG, and the ability of the patients to activate this region during the ‘surrogate’ task of listening-and preparing-to-repeat was used as an index of their ability to activate this region during picture description. A one-way repeated measures ANOVA was used to investigate the effect of session on performance on the picture description test. Mauchley’s test indicated that the assumption of sphericity had been violated, *X*^2^ (2) = 7.3, *P* < 0.05, and therefore the degrees of freedom were corrected using Huynh-Feldt estimates of sphericity (*ε* = 0.76). The results showed that the picture description score was not significantly different between any of the three sessions [*F*(1.5,23) = 1.73, *P* > 0.05]. A one-way repeated measures ANOVA was also conducted to compare the effect of session on the effect size of dACC/SFG activation, which demonstrated that there was no difference between sessions [*F*(2,45) = 0.6, *P* > 0.5] with sphericity assumed. The mean performance on the picture description test across the three sessions was then correlated with the mean dACC/SFG activation across three sessions. There was a significant positive correlation (*r* = 0.63, *P* < 0.01), with better picture description scores associated with greater dACC/SFG activation (Fig. 8).

A multiple regression analysis was used to test if the dACC/SFG activation, age at study and lesion volume significantly predicted participants' picture description score. The results of the regression indicated that the model was statistically significant and accounted for 50% of the variance [*R^2^* = 0.501, *F*(3,12) = 4.02, *P* < 0.03]. It was found that dACC/SFG activation predicted picture description score (*β* = 0.56, *P* < 0.03), but age (*β* = 0.16, *P* < 0.46) and lesion volume did not (*β* = −0.28, *P* = 0.22) (Table 1).

**Table 1:** Multiple regression results

	B	SE B	β
Constant	9.3	25.8	
Mean dACC/SFG	70	27.3	0.56*
Age	0.3	0.4	0.16
Lesion volume	0.0	0.0	−0.28

Results for the multiple regression analysis of the dependent variables mean dACC/SFG activation, age and lesion volume and the dependent variable picture description score. *P < 0.03, R^2 ^= 0.501. B = beta values; SE B = standard error; β = standard error.

## Discussion

This study demonstrated the role of domain-general cognitive control systems in functional imaging studies of language. It has important implications for the interpretation of functional imaging data in patient populations, especially when compared with data from healthy participants. This study also provides evidence for the frequent clinical intuition that impaired function of these systems leads to a poorer prognosis in aphasia.

The imaging analyses on the listening trials performed by the patients separated three broad networks, distributed between the left and right cerebral and cerebellar hemispheres. There was the expected activity in the superior temporal gyri in response to the perception of speech stimuli (Jacquemot *et al.*, 2003; Scott and Wise, 2004; Spitsyna *et al.*, 2006; Warren *et al.*, 2009). However, within the task-dependent context of this study, when participants knew that during the following trial they would be required to repeat back what they had just heard, there was additional activity within areas associated with speech production (Braun *et al.*, 1997; Blank *et al.*, 2002). The predominant distribution was between premotor (medial and lateral) and primary sensorimotor cortices, basal ganglia, thalami and paravermal cerebellum, indicating that motor preparation for the ensuing repetition trial occurred during the listening trial. Additional activity observed in the medial temporal lobes can be attributed to episodic memory encoding of the verbal message.

**Figure 9:**
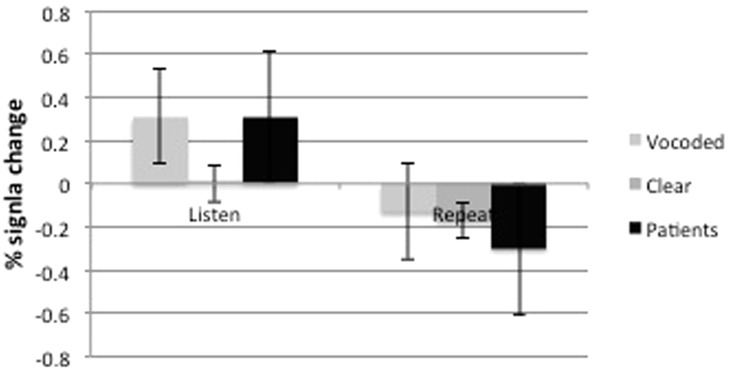
Bar chart, with standard error bars, showing the mean dACC/SFG activation during trials where (*left*) healthy volunteers were listening to ListVoc trials (light grey), listening to ListNorm trials (mid-grey) and patients listening to ListNorm trials (black). *Right*: Also during trials where healthy volunteers were repeating the ListVoc trials (light grey), ListNorm trials (mid- grey) and patients were repeating ListNorm trials (Black).

The third distributed cortical system comprised the cingulo-opercular and dorsolateral prefrontal-parietal networks (salience and central executive networks, respectively). Activity in these networks was revealed in the healthy participants when they listened to three-channel noise-vocoded speech. Therefore, by making listening-and-preparing-to-repeat approximately equal in difficulty for both populations, with similar rates of subsequent repetition success (Fig. 4), the increased activity in domain-general attentional and cognitive control was similar across groups (Fig. 9). One difference was that bilateral basal ganglia signal was evident in the contrast, although the subcortical component of these networks has previously been described as only involving the thalami. The salience and central executive networks are considered to be functionally separable (Dosenbach *et al.*, 2007, 2008), but are usually co-activated as in this study. One proposal is that the central executive network is responsible for moment-to-moment monitoring during the performance of a task, whereas the salience network maintains performance over the time course of repeated trials on that task (Dosenbach *et al.*, 2007). They are functionally connected with cerebellar cortex, activity that was evident in the contrast. The only lateralized cortical component was confined to the posterior left middle and adjacent inferior temporal gyri. This region, based both on lesion and functional imaging studies, has become strongly associated with language-specific processes (Devlin *et al.*, 2000; Hickok and Poppel, 2007; Price, 2010). However, the content of the sentences presented during the clear and noise-vocoded listen trials were semantically and grammatically equivalent. One possibility, therefore, is that the greater left posterior temporal activity during listening to perceptually difficult noise-vocoded speech was the consequence of increased top-down modulation, originating from the salience and central executive networks. This would accord with a role for this region in the controlled access to meaning when perceiving speech (Whitney e*t al*., 2011), with activity increasing as mapping from percept to semantics becomes less automatic with degraded speech stimuli.

Assessing the efficiency of this top-down control in aphasia is not routinely carried out *per se,* not least because linguistic impairments may impact on the accuracy of completing and interpreting formal assessments of cognitive control and vice versa (Fridriksson *et al.*, 2006). However, this functional imaging study has shown intact cognitive control systems become engaged in post-stroke aphasia, in the same manner that it does in healthy participants when the language task was made as difficult by the simple expedient of increasing perceptual difficulty. Therefore, in terms of distributed blood oxygen level-dependency signal, the brain systems responding to task-dependent listening to clear speech in the aphasic patients was similar to that activated in healthy participants when they listened to perceptually difficult noise-vocoded speech. Increased activity in the salience/central executive networks was associated with greater deactivation of the default mode network in both patients and healthy participants. Suppression of the default mode network occurs during goal-directed cognitive processes (Raichle *et al.*, 2001), and the ‘passive’ perception of stimuli or tasks that are habitual or easy to perform on the presented stimuli suppress the default mode network less than tasks that require increased control from executive and attentional networks (Anticevic *et al.*, 2012). Therefore the noise-vocoded speech stimuli, and not the same normal speech stimuli as the patients, elicited most closely the overall activations and deactivations that were observed in the patients with aphasia.

Previous functional imaging studies of aphasic stroke have largely depended on patients responding to or generating verbal information, varying from naming paradigms to other tasks outside the usual common experience, such as verbal fluency (e.g. generating verbs appropriate to an object noun) or word stem completion (e.g. viewing three letters and generating one or more words that incorporate these three initial letters). Although these tasks present healthy participants with a cognitive challenge, there may be a rapid reduction in task-associated activity as the task becomes more familiar or stimuli are repeated (Raichle *et al.*, 1994). In many aphasic participants, the task will prove more challenging and task habituation may occur more slowly in the face of increased difficulty because of the presence of the lesion. It can be predicted from the present study that these tasks will also involve activation of domain-general salience and central executive networks, in addition to language-specific systems. Most studies have related the results in patients to healthy participants performing exactly the same stimuli and task as the patients. One temptation has been to suggest that right cerebral hemisphere activity in the patient group relative to the normal group, particularly when it is in or close to what might be regarded as the right hemisphere homologue of Broca’s area, is a shift in the lateralization of language-specific processes. The results from this study, in which the strategy has been to increase task difficulty for the healthy participants and reduce their in-scanner task performance to the level of the patients, suggest that the previous studies were observing upregulation of normal domain-general cognitive control systems in the patients as they attempted a task that was unusually difficult for them as the consequence of their stroke (Rosen *et al.*, 2000; Wise, 2003).

The analysis of this study then turned to whether the function of a central component of the combined domain-general salience and central executive networks reflected language recovery. The dACC/SFG was chosen as it lies in anterior cerebral artery territory, and therefore outside middle cerebral artery territory in which the aphasic strokes had occurred. This region was macroscopically intact in all patients. Activating the dACC/SFG with one task (‘listen-and-prepare-to-repeat’), in the knowledge that self-generated speech also activates this region, motivated the analysis correlating its function with the patients’ out-of-scanner performance on a widely used and ecologically valid assessment of speech production in aphasia, namely picture description. The result demonstrated that in chronic aphasic patients the activation of the dACC/SFG predicted performance on this test. This correlation did not change when a multiple regression analysis was performed that included the volume of infarction and the ages of the patients. Although the in-scanner task and the picture description task required different input and output systems, the activation in the dACC reflects increased task difficulty regardless of whether the specific language task emphasizes speech comprehension or production. Therefore, the role of the dACC is not specific to one of the two broad divisions applied to language, namely ‘receptive’ or ‘expressive, but to the cognitive control of language processing in general.

Although the importance of both particular lesion location, irrespective of total volume, and impairment of particular language processes, will undoubtedly account for some of the variance observed in language recovery, this study has demonstrated that the function of domain-general cognitive control systems also has a significant impact on recovery. This study was not designed to determine why the dACC/SFG had such variable function across the group. In addition to a remote effect of long fibre tract infarction, the microscopic effects of disease predisposing to stroke (such as hypertension and diabetes) and biological (which is not necessarily the same as chronological) ageing are probable factors influencing dACC/SFG function. Future studies could incorporate metabolic and neuroligand PET studies of this region, coupled with diffusion tensor MRI of white matter tracts, to investigate these possibilities.

The healthy participants responded to 2 weeks of training on the noise-vocoded sentences and showed a significant improvement in their ability to perceive and repeat these sentences. The behavioural therapy on the patients over 4 weeks resulted in a significant improvement in speech perception, as indexed by improved word same/different discrimination tests that could be attributed to the training and not to practice effects. This behavioural result has been submitted for publication elsewhere. However, their ability to repeat did not improve, and this probably reflects parallel damage to posterior-anterior speech production pathways, which were not the target of the behavioural therapy. Furthermore, their proficiency at online repetition during the three scanning sessions did not improve. Despite the specific (perception of phonological distinctions) responses to training, there was no evident functional imaging correlate in the contrasts between pre- and post-training image data in either the healthy participants or the patients. This study reports conventional univariate statistical analyses, which may be too insensitive to reveal the training–induced functional changes. Further analyses using more sensitive multivariate techniques may be required. It will also be an advantage to recruit more patients, although this may need the participation of multiple centres. Only a minority of patients are eligible (Supplementary material), and subgroup analyses, planned in advance, may be required if lesion location and volume and behavioural deficit are heterogenous, which will add noise to overall group analyses.

In summary, this study has demonstrated the role of domain-general cognitive control systems in language tasks, and the potential influence of their activation on the interpretation of functional imaging data in patient populations. More importantly, this study has indicated that impaired function of these systems has an impact on final outcome, a non-language impairment that might usefully be a target for rehabilitative techniques in addition to any therapy that targets language-specific processes.

## Funding

All participants gave informed consent to participate in the study. The study was funded by the Wellcome trust (079098/Z/06/Z) and Medical Research Council and the Royal College of Surgeons (G0802270).

## Supplementary Material

Supplementary Data

## Abbreviations

dACC/SFG: dorsal anterior cingulate cortex and adjacent part of the midline superior frontal gyrus
ListTrials: listen-and-prepare-to-repeat trials
ListVoc: listen to vocoded stimuli-and-prepare-to-repeat trials
ListWhite: listen to white noise
ListAll: listen to vocoded and normal stimuli-and-prepare-to-repeat trials

## References

[awt289-B1] Abo M, Senoo A, Watanabe S, Miyano S, Doseki K, Sasaki N (2004). Language-related brain function during word repetition in post-stroke aphasics. Neuroreport.

[awt289-B2] Anticevic A, Cole MW, Murray JD, Corlett PR, Wang XJ, Krystal JH (2012). The role of default network deactivation in cognition and disease. Trends Cogn Sci.

[awt289-B3] Bakheit AM, Shaw S, Carrington S, Griffiths S (2007). The rate and extent of improvement with therapy from the different types of aphasia in the first year after stroke. Clin Rehabil.

[awt289-B4] Bench J, Kowal A, Bamford J (1979). The BKB (Bamford-Kowal-Bench) sentence lists for partially- hearing children. Br J Audiol.

[awt289-B5] Blank SC, Bird H, Turkheimer F, Wise RJ (2003). Speech production after stroke: the role of the right pars opercularis. Ann Neurol.

[awt289-B6] Blank SC, Scott SK, Murphy K, Warburton E, Wise RJ (2002). Speech production: Wernicke, Broca and beyond. Brain.

[awt289-B7] Bonnelle V, Ham TE, Leech R, Kinnunen KM, Mehta MA, Greenwood RJ (2012). Salience network integrity predicts default mode network function after traumatic brain injury. Proc Natl Acad Sci USA.

[awt289-B8] Braun AR, Varga M, Stager S, Schulz G, Selbie S, Maisog JM (1997). Altered patterns of cerebral activity during speech and language production in developmental stuttering. An H2(15)O positron emission tomography study. Brain.

[awt289-B9] Brett M, Leff AP, Rorden C, Ashburner J (2001). Spatial normalization of brain images with focal lesions using cost function masking. Neuroimage.

[awt289-B70] Devlin JT, Russell RP, Davis MH, Price CJ, Wilson J, Moss HE, Matthews PM, Tyler LK (2000). Susceptibility-induced loss of signal: comparing PET and fMRI on a semantic task. Neuroimage.

[awt289-B10] Dosenbach NU, Fair DA, Cohen AL, Schlaggar BL, Petersen SE (2008). A dual-networks architecture of top-down control. Trends Cogn Sci.

[awt289-B11] Dosenbach NU, Fair DA, Miezin FM, Cohen AL, Wenger KK, Dosenbach RA (2007). Distinct brain networks for adaptive and stable task control in humans. Proc Natl Acad Sci USA.

[awt289-B12] Fedorenko E, Duncan J, Kanwisher N (2012). Language-selective and domain-general regions lie side by side within Broca's area. Curr Biol.

[awt289-B13] Fernandez B, Cardebat D, Demonet JF, Joseph PA, Mazaux JM, Barat M (2004). Functional MRI follow-up study of language processes in healthy participants and during recovery in a case of aphasia. Stroke.

[awt289-B15] Fridriksson J, Morrow L (2005). Cortical activation and language task difficulty in aphasia. Aphasiology.

[awt289-B16] Fridriksson J, Nettles C, Davis M, Morrow L, Montgomery A (2006). Functional communication and executive function in aphasia. Clin Linguist Phon.

[awt289-B17] Geranmayeh F, Brownsett SL, Leech R, Beckmann CF, Woodhead Z, Wise RJ (2012). The contribution of the inferior parietal cortex to spoken language production. Brain Lang.

[awt289-B18] Greicius MD, Krasnow B, Reiss AL, Menon V (2003). Functional connectivity in the resting brain: a network analysis of the default mode hypothesis. Proc Natl Acad Sci USA.

[awt289-B19] Greicius MD, Menon V (2004). Default-mode activity during a passive sensory task: uncoupled from deactivation but impacting activation. J Cogn Neurosci.

[awt289-B20] Hamilton RH, Chrysikou EG, Coslett B (2011). Mechanisms of aphasia recovery after stroke and the role of noninvasive brain stimulation. Brain Lang.

[awt289-B21] Heiss WD, Kessler J, Thiel A, Ghaemi M, Karbe H (1999). Differential capacity of left and right hemispheric areas for compensation of poststroke aphasia. Ann Neurol.

[awt289-B22] IBM Corp IBM SPSS statistics for windows, version 20.0. Released 2011.

[awt289-B69] Hickok G, Poeppel D (2007). The cortical organization of speech processing. Nat Rev Neurosci.

[awt289-B23] Hula WD, McNeil MR (2008). Models of attention and dual-task performance as explanatory constructs in aphasia. Semin Speech Lang.

[awt289-B24] Jacquemot C, Pallier C, LeBihan D, Dehaene S, Dupoux E (2003). Phonological grammar shapes the auditory cortex: a functional magnetic resonance imaging study. J Neurosci.

[awt289-B25] Kertesz A, Harlock W, Coates R (1979). Computer tomographic localization, lesion size, and prognosis in aphasia and nonverbal impairment. Brain Lang.

[awt289-B26] Kertesz A, McCabe P (1977). Recovery patterns and prognosis in aphasia. Brain.

[awt289-B27] Lazar RM, Speizer AE, Festa JR, Krakauer JW, Marshall RS (2008). Variability in language recovery after first-time stroke. J Neurol Neurosurg Psychiatry.

[awt289-B28] Leff A, Crinion J, Scott S, Turkheimer F, Howard D, Wise R (2002). A physiological change in the homotopic cortex following left posterior temporal lobe infarction. Ann Neurol.

[awt289-B29] Lendrem W, Lincoln NB (1985). Spontaneous recovery of language in patients with aphasia between 4 and 34 weeks after stroke. J Neurol Neurosurg Psychiatry.

[awt289-B30] Lovstad M, Funderud I, Meling T, Kramer UM, Voytek B, Due-Tonnessen P (2012). Anterior cingulate cortex and cognitive control: neuropsychological and electrophysiological findings in two patients with lesions to dorsomedial prefrontal cortex. Brain Cogn.

[awt289-B31] Meinzer M, Harnish S, Conway T, Crosson B (2011). Recent developments in functional and structural imaging of aphasia recovery after stroke. Aphasiology.

[awt289-B32] Menon V, Uddin LQ (2010). Saliency, switching, attention and control: a network model of insula function. Brain Struct Funct.

[awt289-B33] Mimura M, Kato M, Kato M, Sano Y, Kojima T, Naeser M (1998). Prospective and retrospective studies of recovery in aphasia. Changes in cerebral blood flow and language functions. Brain.

[awt289-B35] Musso M, Weiller C, Kiebel S, Muller SP, Bulau P, Rijntjes M (1999). Training-induced brain plasticity in aphasia. Brain.

[awt289-B36] Naeser MA, Martin PI, Baker EH, Hodge SM, Sczerzenie SE, Nicholas M (2004). Overt propositional speech in chronic nonfluent aphasia studied with the dynamic susceptibility contrast fMRI method. Neuroimage.

[awt289-B37] Naeser MA, Martin PI, Nicholas M, Baker EH, Seekins H, Kobayashi M (2005). Improved picture naming in chronic aphasia after TMS to part of right Broca's area: an open-protocol study. Brain Lang.

[awt289-B38] Novick JM, Kan IP, Trueswell JC, Thompson-Schill SL (2009). A case for conflict across multiple domains: memory and language impairments following damage to ventrolateral prefrontal cortex. Cogn Neuropsychol.

[awt289-B39] Novick JM, Trueswell JC, Thompson-Schill SL (2010). Broca’s area and language processing: evidence for the cognitive control connection. Lang Linguist Compass.

[awt289-B41] Pedersen PM, Vinter K, Olsen TS (2004). Aphasia after stroke: type, severity and prognosis. Cerebrovasc Dis.

[awt289-B42] Plowman E, Hentz B, Ellis C (2012). Post-stroke aphasia prognosis: a review of patient-related and stroke-related factors. J Eval Clin Pract.

[awt289-B73] Price CJ (2010). The anatomy of language: a review of 100 fMRI studies published in 2009. Ann N Y Acad Sci.

[awt289-B43] Price C, Crinion J (2005). The latest on functional imaging studies of aphasic stroke. Curr Opin Neurol.

[awt289-B44] Price C, Friston K (1999). Scanning patients with tasks they can perform. Hum Brain Mapp.

[awt289-B46] Raboyeau G, De Boissezon X, Marie N, Balduyck S, Puel M, Bezy C (2008). Right hemisphere activation in recovery from aphasia: lesion effect or function recruitment?. Neurology.

[awt289-B47] Raichle ME, Fiez JA, Videen TO, MacLeod AM, Pardo JV, Fox PT (1994). Practice-related changes in human brain functional anatomy during nonmotor learning. Cerebral Cortex.

[awt289-B48] Raichle ME, MacLeod AM, Snyder AZ, Powers WJ, Gusnard DA, Shulman GL (2001). A default mode of brain function. Proc Natl Acad Sci USA.

[awt289-B49] Richter M, Miltner WH, Straube T (2008). Association between therapy outcome and right-hemispheric activation in chronic aphasia. Brain.

[awt289-B50] Rosen HJ, Petersen SE, Linenweber MR, Snyder AZ, White DA, Chapman L (2000). Neural correlates of recovery from aphasia after damage to left inferior frontal cortex. Neurology.

[awt289-B52] Saur D, Lange R, Baumgaertner A, Schraknepper V, Willmes K, Rijntjes M (2006). Dynamics of language reorganization after stroke. Brain.

[awt289-B53] Saur D, Ronneberger O, Kummerer D, Mader I, Weiller C, Kloppel S (2010). Early functional magnetic resonance imaging activations predict language outcome after stroke. Brain.

[awt289-B54] Schofield TM, Penny WD, Stephan KE, Crinion JT, Thompson AJ, Price CJ (2012). Changes in auditory feedback connections determine the severity of speech processing deficits after stroke. J Neurosci.

[awt289-B71] Scott S, Wise R (2004). The functional neuroanatomy of prelexical processing in speech perception. Cognition.

[awt289-B55] Shannon RV, Zeng FG, Kamath V, Wygonski J, Ekelid M (1995). Speech recognition with primarily temporal cues. Science.

[awt289-B56] Sharp DJ, Scott SK, Wise RJ (2004). Retrieving meaning after temporal lobe infarction: the role of the basal language area. Ann Neurol.

[awt289-B57] Snyder HR, Feigenson K, Thompson-Schill SL (2007). Prefrontal cortical response to conflict during semantic and phonological tasks. J Cogn Neurosci.

[awt289-B58] Spitsyna G, Warren J, Scott S, Turkheimer F, Wise R (2006). Converging language streams in the human temporal lobe. J Neurosci.

[awt289-B59] Swinburn K, Porter G, Howard D (2004). Comprehensive aphasia test.

[awt289-B60] Thiel A, Herholz K, Koyuncu A, Ghaemi M, Kracht LW, Habedank B (2001). Plasticity of language networks in patients with brain tumors: a positron emission tomography activation study. Ann Neurol.

[awt289-B72] Thompson CK, Fix S, Gitelman DG, Parrish TB, Mesulam MM (2000). fMRI studies of agrammatic sentence comprehension before and after treatment. Brain and Language.

[awt289-B62] Tseng C-H, McNeil MR, Milenkovic P (1993). An investigation of attention allocation deficits in aphasia. Brain Lang.

[awt289-B63] Warburton E, Price CJ, Swinburn K, Wise RJ (1999). Mechanisms of recovery from aphasia: evidence from positron emission tomography studies. J Neurol Neurosurg Psychiatry.

[awt289-B64] Warren JE, Crinion JT, Lambon Ralph MA, Wise RJS (2009). Anterior temporal lobe connectivity correlates with functional outcome after aphasic stroke. Brain.

[awt289-B65] Weiller C, Isensee C, Rijntjes M, Huber W, Muller S, Bier D (1995). Recovery from Wernicke's aphasia: a positron emission tomographic study. Ann Neurol.

[awt289-B66] Whitney C, Kirk M, O'Sullivan J, Lambon Ralph MA, Jefferies E (2011). The neural organization of semantic control: TMS evidence for a distributed network in left inferior frontal and posterior middle temporal gyrus. Cereb Cortex.

[awt289-B67] Winhuisen L, Thiel A, Schumacher B, Kessler J, Rudolf J, Haupt WF (2007). The right inferior frontal gyrus and poststroke aphasia: a follow-up investigation. Stroke.

[awt289-B68] Wise RJ (2003). Language systems in normal and aphasic human participants: functional imaging studies and inferences from animal studies. British Medical Bulletin.

