# Supplementary material for: Cognitive control and its impact on recovery from aphasic stroke
Source: Brain. 2013 Oct 24;137(1):242–54. doi: 10.1093/brain/awt289 (PMC3891442; doi:10.1093/brain/awt289)
Supplement: Supplementary Data [file supp_137_1_242__index.html]

Cognitive control and its impact on recovery from aphasic stroke — Supplementary Data 

# Cognitive control and its impact on recovery from aphasic stroke

## Supplementary Data

files

**Files in this Data Supplement:**

- Supplementary Data - docx file
