# Supplementary material for: Cognitive control and its impact on recovery from aphasic stroke
Source: Brain. 2013 Oct 24;137(1):242–54. doi: 10.1093/brain/awt289 (PMC3891442; doi:10.1093/brain/awt289)
Supplement: Supplementary Data [file supp_awt289_brain-2013-00991-File012.docx]

**Supplementary Methods**

Nineteen patients did not wish to be included in the study, a further 19 had severe co-morbid disease, 12 had English as an additional language and seven were unable to give informed consent due to the severity of their impairment. All participants were required to undergo a MRI study to locate their infarct and to exclude the presence of other lesions (e.g. lobar infarcts in the contralateral hemisphere). Eight patients were excluded due to contraindications to MRI, and four only agreed to have behavioural but not scanning assessments. Two further patients withdrew from the study after attempting the first scan. One participant was excluded from analyses because the severity of his dysarthria did not allow reliable scoring of his in-scanner responses or picture description performance.

A total of 21 healthy participants were recruited for the study. Two participants did not complete the training programme or the second scan, and two were excluded due to abnormal findings on their anatomical scan.
